# How Leader-Member Exchange Affects Knowledge Sharing Behavior: Understanding the Effects of Commitment and Employee Characteristics

**DOI:** 10.3389/fpsyg.2019.02768

**Published:** 2019-12-10

**Authors:** Qi Hao, Yijun Shi, Weiguo Yang

**Affiliations:** ^1^The School of Labor and Human Resources, Renmin University, Beijing, China; ^2^The School of Foreign Languages, Renmin University, Beijing, China

**Keywords:** leader-member exchange, affective commitment, general self-efficacy, internal locus of control, knowledge sharing behavior

## Abstract

Although leadership is considered a key factor in affecting employees’ knowledge sharing behavior (KSB), previous literature has mainly focused on the direct relationship between it and KSB, neglecting the mediators and moderators in this relationship. To address this issue, this study explores when and how leader-member exchange (LMX) promotes KSB by examining affective commitment (AC) as mediator and employee general self-efficacy (GSE) and internal locus of control (ILOC) as boundary conditions. In addition, although these two positive self-view variables (i.e., GSE and ILOC) both exhibit positive effects on various work-related outcomes, based on self-verification theory, we posit that they may exhibit different moderating effects in the LMX–AC–KSB relationship. We empirically validated this moderated mediated model using data collected from 231 supervisor–subordinate pairs from an information technology company in China. The results show that GSE amplifies the mediated relationship between LXM and KSB via AC, but ILOC weakens this mediated relationship. Our study elucidates when and how LMX can effectively facilitate KSB and sheds new and nuanced light on the conceptual distinction between GSE and ILOC. The results of this study might direct managers how to develop relationships with their subordinates and how to maximally facilitate subordinates’ KSB.

## Introduction

Organizational knowledge can help organizations underpin competitive advantages and is difficult to imitate or replaced by third parties (Cabrera et al., 2006). Thus, it is considered a worthy, scarce, and highly strategic resource which deserves a great deal of managers’ diligent attention (Lee et al., 2018; Hao et al., 2019). In the past few decades, various knowledge management systems or technologies have been designed to facilitate knowledge sharing among employees (Cabrera et al., 2006). However, scholars are gradually realizing that the major barriers preventing companies from effectively managing knowledge reside in people rather than in technologies (Lin, 2007; Pee and Min, 2017; Lee et al., 2018). People resist sharing their expertise because knowledge sharing behavior (KSB) usually demands high costs from and imposes risks on them, which may put them in a situation called the KSB dilemma (Cabrera and Cabrera, 2002; Ardichvili et al., 2003; Pee and Lee, 2015). On the one hand, when sharing knowledge, people need to convert their specialized knowledge and unique skills into an understandable and applicable form for the receivers, and this process may take more time and energy (Pee and Lee, 2015; Hao et al., 2019). On the other hand, as the saying “possession is nine-tenths of the law” indicates, employees may not elect to share their idiosyncratic thoughts and experiences with colleagues to keep their individual power and competitive advantages (Jeung et al., 2017; Lee et al., 2018). In this regard, stimulating employees’ KSB is considered a challenging job, unless the sharing process can generate greater benefits, such as in an individual benefit, i.e., self-interest, personal gain (Wasko and Faraj, 2000; Pee and Lee, 2015); a group benefit, i.e., reciprocal behaviors, relationships with others (Ko et al., 2005; Chae et al., 2015); or an organizational benefit, i.e., organizational gain, organizational support (Bock et al., 2005; Jeung et al., 2017).

According to this principle, a number of studies on knowledge sharing that draw on the exchange and reciprocity theories, such as the leader–member exchange (LMX) theory (Graen et al., 1977), have occurred. In fact, researchers have long been interested in how leadership can affect KSB. Different types of leadership or different levels of LMX might show different effects on employees’ KSB. For example, some researchers found that empowering leadership can effectively facilitate employees’ KSB by positively affecting their attitudes toward KSB (Xue et al., 2011). Liu and Li (2018) found that perceived team goal commitment and perceived team identification both mediate the positive relationship between transformational leadership and KSB. Lee et al. (2018) study revealed a negative relationship between abusive supervision and KSB. Some researchers also found that different levels of LMX differently affect employees’ KSB (Su et al., 2013). Despite the growing body of studies on this issue, there is unresolved ambiguity about the nature of this relationship (Carmeli et al., 2013). First, most researchers have focused solely on the direct correlation between LMX and KSB (Kim et al., 2017), neglecting the intermediate psychological processes underlying the relationship. Second, although high-quality LMX may cultivate a favorable social context for employees (Carmeli et al., 2011), different people may evaluate this situation in different manners (Kim et al., 2017), echoing the interactionist approach, in which personal characteristics and contextual factors jointly affect individual’s behaviors (e.g., Abbas et al., 2015; Pee and Min, 2017; Lee et al., 2018). Third, most previous studies on the relationship between leadership and employee outcomes are established and widely practical in the western context (Law et al., 2000). Some scholars contended that these motivation models may not work equally in the Chinese culture (e.g., Hofstede, 1993). Particularly, in China, there is a special form of interpersonal relationship between leaders and followers called workplace *guanxi*, referring to interpersonal bonds that can create specific expectations and duties (Law et al., 2000). *Guanxi* plays an important role in affecting the exchange of personal resources and information in China (Wang et al., 2012). Thus, it is crucial to uncover the complex mechanism underlying the LMX–KSB relationship in the Chinese context.

In the current study, we select affective commitment (AC) as a mediator and two individual characteristics [i.e., general self-efficacy (GSE) and internal locus of control (ILOC)] as moderators. The rationale for selecting AC as a mediator is twofold: First, AC is the most analyzed form of organizational commitment (Gaudet and Tremblay, 2017), and it has already been considered a mediator in explaining the relationship between leadership and employee behaviors in many studies (e.g., Chang et al., 2015; Gupta et al., 2016; Gaudet and Tremblay, 2017; Jeung et al., 2017). Second, the commonly accepted job experience-attitude-behavior sequence (Zhao et al., 2007; Gupta et al., 2016) shows that positive work experiences (such as high-quality LMX) are viewed as affective events, and affective reactions (such as AC) that lead to effectiveness outcomes (such as KSB) are the proximal consequences of these experiences. This argument indicates that AC might be a suitable mediator in explaining the LMX–KSB relationship.

The current study assigns GSE and ILOC as personal moderators for three reasons. First, there has been sufficient research on the relationship between Big-Five personality and KSB (e.g., Barrick and Mount, 1991; Hao et al., 2019), but little attention has been assigned on how the other personality constructs, such as core self-evaluation traits, affect KSB (Judge and Bono, 2001). Second, GSE and ILOC are two key elements of the core self-evaluations traits (Judge et al., 1997). Many studies have demonstrated that these variables can affect individuals’ reactions to different leader behaviors (e.g., Ehrhart and Klein, 2001; Chen et al., 2016). Third, according to self-verification theory (Swann, 2011), although GSE and ILOC are positively related and demonstrate similar effects on various outcomes (Judge et al., 1997; Judge and Bono, 2001), they “orient people toward different aspects of the information embedded in the [same] context: competence for [GSE] and source of influence over personal outcomes for [ILOC]” (Chen et al., 2016, p. 125). In this regard, these two similar constructs may result in distinct reactions to the effects of LMX which piques our interest in exploring the different moderating effects of GSE and ILOC on the LMX-KSB relationship.

**FIGURE 1:**
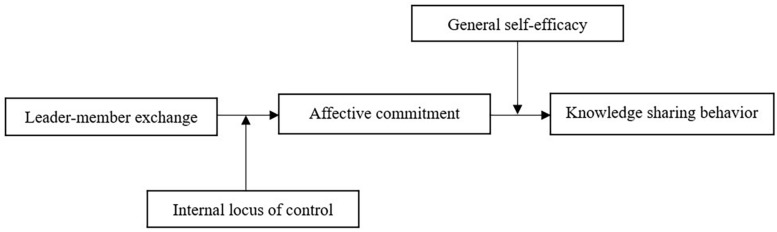
Research model.

To simultaneously uncover the complex mechanism underlying the LMX–KSB relationship and to further advance the theories about the joint effects of contextual factors and personal characteristics, we develop a theoretical model in which diverse employee characteristics differentially moderate the influence of LMX. Specifically, the current study examines the mediating effects of AC and the different moderating roles of GSE and ILOC (see Figure 1). Our research may contribute to the exist literature in the following three ways: First, this study elucidates when and how LMX can effectively facilitate KSB; that is, examining if KSB could be a result of employees’ increasing AC induced by the high-quality LMX they experienced. Second, our study extends the person-context interactionist perspective by exploring the different moderating effects of two personal characteristics (i.e., GSE and ILOC) on the LMX–AC–KSB relationship. Third, the current study sheds new and nuanced light on the conceptual distinction between GSE and ILOC – two highly parallel, core self-evaluations variables.

## Theory and Hypotheses

### LMX and KSB

Knowledge sharing behavior is defined as individuals transforming their work-relevant ideas, experience and suggestions into understandable and applicable forms for the knowledge receivers (Hao et al., 2019; Kim, 2019). In work settings, KSB can be considered one of the extra-role behaviors such as organizational citizenship behavior (OCB) because these behaviors are not formally prescribed by organizations, difficult to measure, and problematic to formally appraise (Love and Forret, 2008; Edú-Valsania et al., 2016). However, KSB often demands higher costs from or poses greater risks to individuals than other discretionary behaviors. First, KSB goes beyond the simple communication of information and representation of tasks and procedural message (Carmeli et al., 2011). Rather, it is a process involving sharing, teaching, and learning, which may cost individuals’ valuable time that might be used in other tasks (Jeung et al., 2017). Second, sharing their specialized knowledge and unique skills may make people less competitive (Kim et al., 2017). In this regard, people may refuse to share their unique expertise with others, despite its contribution in enhancing organizational competitiveness (Lee et al., 2018). To solve this issue, some scholars point out that leaders in organizations are in positions to help their followers overcome this resistance (Carmeli et al., 2011). They argue that leaders can cultivate a social context in which employees can not only obtain sufficient KSB mentoring but also effectively improve their sharing intentions (Carmeli et al., 2011).

One of these social contexts is LMX, which is defined as “the dyadic exchange relationship between supervisors and employees” within an organizational work unit (Kim et al., 2017, p. 152). Graen et al. (1977) pioneered the introduction of LMX using role-playing theory. Later, some scholars studied LMX based on the reciprocity continuum (Schriesheim et al., 1999). Recently, scholars are focusing on the social exchange perspectives (e.g., Casimir et al., 2014; Kim et al., 2017), in which LMX relationships are grossly divided into two categories: “low-quality” and “high-quality.” Furthermore, the quality of LMX depends on how leaders interact with their followers. Low-quality LMX appears when leaders and their followers rarely communicate with and distrust each other whereas high-quality LMX occurs when there is a social exchange between leaders and employees; that is, the exchange happens beyond the employment contract (Graen et al., 1977; Graen and Uhl-Bien, 1995; Casimir et al., 2014). In a high-quality LMX relationship, a mutually trustworthy, motivated, and favorable climate can be constructed between leaders and employees. In addition, an employee who experiences high-quality LMX usually involves in more decision-making processes, fewer task-related problems, and is more incline to undertake organizational responsibilities (Casimir et al., 2014). Thus, high-quality LMX may help employees generate positive work experience, prompting them to go beyond requirements and to exhibit more voluntary behaviors, such as KSB (Casimir et al., 2014; Kim et al., 2017). Scholars also suggest that high-quality LMX can stimulate subordinates to internalize organizational goals; in other words, employees will focus on collective benefits rather than to individual benefits (Carmeli et al., 2011; Su et al., 2013). Thus, the risks and costs accompanying KSB will be alleviated, which, in turn, elevates KSB.

The above arguments are reflected in previous studies. For example, Li et al. (2014) argued that high-quality LMX may make workers feel committed, loyal and collectivistic, which leads to higher-levels of KSB. Some researchers also stated that in order to obtain desired outcomes from high-quality LMX, followers may pay more attention to the interests of the collective, which may facilitate them to perform more beneficial behaviors, such as KSB (Sharifkhani et al., 2016). In addition, Anand et al. (2018) suggested that employees may reciprocate their leaders’ favorable treatment by performing more discretionary behaviors, such as courtesy behaviors, altruistic behaviors or helping behaviors. Taking all this together, we hypothesize that:

*H1*: LMX is positively related to KSB.

### The Mediating Role of AC

Affective commitment, which is defined as employees’ emotional attachment to, identification with, and involvement in an organization (Meyer and Allen, 1991), has become the most analyzed form of organizational commitment (Gaudet and Tremblay, 2017). A high sense of commitment to an organization usually helps an employee identify with this company’s core values and main goals (Casimir et al., 2014). Through this identification process the employee can generate proud feeling of being part of this organization. Employees’ emotional attachment to an organization can be enhanced by numerous factors. For instance, organizational justice (Karriker and Williams, 2009), job designs (Currivan, 1999), supportive leadership (Joo, 2010), and intrinsic and extrinsic rewards (O’Driscoll and Randall, 1999) have all been found to positively affect employees’ AC. Among these commonly identified antecedents, the one most strongly associated with AC may be leadership, especially high-quality LMX (Casimir et al., 2014; Jeung et al., 2017; Curtis and Taylor, 2018).

Scholars explain the LMX–AC relationship by the following two lines of theories: First, employees with high-quality LMX tend to obtain more emotional and material support from their leaders and organizations than the others (Jeung et al., 2017). This positive treatment may create a feeling among subordinates of an obligation to pay back the favorable treatment they have received. Drawing on the social exchange theory (Blau, 1964) and the promise of reciprocation (Gouldner, 1960), employees will satisfy their indebtedness by generating a greater emotional bond with the organization. Second, high-quality LMX meet various socioemotional needs of employees, such as affiliation, esteem, approval, and emotional support, thereby creating favorable working conditions (Rhoades et al., 2001). In this case, employees prefer to incorporate organizational membership and role status into their social identities, generate a feeling of belonging to the organization, and foster emotional attachment to the organization (Casimir et al., 2014; Jeung et al., 2017). In line with these theories, we assume that high-quality LMX contributes to facilitating AC.

In nature, KSB is a voluntary activity that is fundamentally unobservable by others (Curtis and Taylor, 2018). Therefore, the organization usually cannot impose external controls on employees or require them to share their knowledge. In this regard, individuals share their valuable expertise only when they are willing to do so, to benefit others or the organization (Jeung et al., 2017). Individuals who have high levels of AC tend to view the organization as an extended family and the organization’s problems as their own (Meyer and Herscovitch, 2001; Casimir et al., 2014). As a result, a great sense of commitment to an organization can help to overcome the KSB dilemma, as individuals pay more attention to the goals of the organization and the collective welfare of other members rather than emphasizing solely on their own costs and benefits (Cabrera and Cabrera, 2002; Pee and Lee, 2015). Furthermore, some scholars also state that when individuals build strong emotional bonds with an organization, they may even believe that the organization has the right to their knowledge (Jarvenpaa and Staples, 2001). In support of these arguments, previous studies have consistently demonstrated positive relationship between AC and KSB. For example, from a commitment-trust theory perspective, Hashim and Tan (2015) demonstrated that an individual’s commitment to his/her organization positively affects his/her continuous knowledge sharing intention. Van Den Hooff and De Ridder (2004) stated that AC is an essential part of a knowledge sharing culture. Moreover, some scholars argued that attitudinal predictors, such as AC, were considered the most consistent factors facilitating employees’ OCB or other extra-role behaviors (Ng and Feldman, 2011).

Following these studies, the current study assumes that employees experiencing high-quality LMX can develop stronger emotional attachments to the organization. Consequently, they are incline to share their expertise with colleagues to help the organization, thereby promoting its effectiveness. In other words, the present study implicitly constructs a model in which AC plays mediated role in the LMX–AC relationship. Thus, we posit the following hypothesis:

*H2*: The relationship between LMX and KSB is mediated by AC.

### The Moderating Role of GSE and ILOC

To further investigate the complex mechanism between LMX and KSB, we draw on the person–context interactionist perspective (Pee and Min, 2017; Hao et al., 2019), to explore the moderating effects of employee characteristics. Judge et al. (1997) proposed a higher order construct that they termed “core self-evaluations traits,” defined as a fundamental appraisal of one’s effectiveness, worthiness, and capability as a person. This construct includes four well-established traits (Judge et al., 1997): self-esteem, neuroticism, GSE, and ILOC. Among these, the present study focuses on GSE and ILOC – two conceptually similar constructs, both reflecting individuals’ belief that they are in control of their own success (Chen et al., 2016). Despite their common ground, GSE and ILOC emphasize different aspects: that is, GSE highlights individuals’ belief in their capacities in dealing with various tasks (Bandura, 1989, 1997), whereas ILOC focuses on the belief that internal factors (e.g., tenacity, effort, and talent), instead of external elements (e.g., environment, luck, and help from others), determine their performance (Rotter, 1966).

According to self-verification theory (Swann, 2011), individuals are strongly motivated to accept experiences that consistent with their preconceived notions, and to avert the disconfirmation experiences. Bosson and Swann (1999) argued that different types of positive self-views may related to different reactions. GSE and ILOC represent different types of positive self-views. GSE is a type of self-competence variable which is related to self-competence feedback, whereas ILOC represents a type of self-liking variable that is related to self-liking feedback. In this regard, we propose that people with high levels of GSE or ILOC may focus on different aspects of the information embedded in LMX and generate different kinds of feedback. These different self-verification processes can lead to different moderating effects for GSE and ILOC.

#### The Positive Moderating Effect of GSE on LMX–AC–KSB

As we have posited, high-quality LMX can enhance employees’ emotional bonds with the organization, which in turn creates a strong “reason to” exhibit more KSB. However, some scholars argued that only having a “reason to” share knowledge is not enough. There is another pivotal determinant for KSB: a “can do” attitude, or an individual’s feeling of being able to perform such behavior (e.g., Hsu et al., 2007; Raub and Liao, 2012). They further suggest that fundamental to such a “can do” attitude is GSE.

General self-efficacy, which is considered a relatively stable, idiosyncratic construct (Pan et al., 2011), refers to an individual’s belief in his/her overall competence or ability to perform across a variety of situations (Judge et al., 1998). Drawing on Bandura’s (1997) theory, an individual’s choice behaviors, feelings of stress and anxiety, efforts to overcome problems, and job performance are all influenced by GSE. Here, we predict that the “can do” factor – namely, GSE – can interact with and strengthen the positive effect of the “reason to” factor. Thus, despite individuals develop strong emotional attachments to the organization and sincerely want to perform more discretionary activities, if the “can do” factors are missing – that is, if they doubt their ability to execute such activities successfully, individuals may not likely to proactively exhibit such discretionary behaviors, particularly KSB. Accordingly, the effects of AC on KSB will be significantly weakened for employees with lower GSE. On the contrary, potential knowledge contributors with higher GSE tend to feel less anxious and more competent and confident than individuals with lower GSE (Pan et al., 2011). Self-verification theory suggests that self-efficacious individuals are more attentive to other motivational factors and respond to them more positively in terms of exhibiting more interest in helping the organization succeed. Consistent with this view, employees who are highly involved in and identify with the organization, coupled with their higher GSE, will exhibit as much KSB as they can. In other words, we predict that the effect of AC on KSB should be stronger for self-efficacious individuals. Thus, we propose the following hypothesis:

*H3a*: Employee’ GSE moderates the positive relationship between AC and KSB, such that the higher the level of GSE, the stronger the relationship.

According to the previous literature, if a moderator alters the path from an independent variable to a mediator or the path from a mediator to the dependent variable, that same moderator then impacts the entire mediated relationship (Edwards and Lambert, 2007). In our case, the significant moderation of the link between AC and KSB by GSE, together with the mediated relationship between LMX and KSB via AC, a moderated mediation model thus arises typically.

As already explained, self-efficacious subordinates are more likely to perceive the positive psychological situations created by high-quality LMX and react more positively. Thus, high-quality LMX is more effective in stimulating these employees to contribute more KSB by elevating their emotional bonds with the organization. In this regard, AC plays a more important mediating role in transmitting the effect of LMX on KSB for employees high in GSE. Conversely, individuals who have low GSE are less attentive to favorable treatments or psychological situations (Giesler et al., 1996). They may not exhibit as much KSB as expected even when they build a strong emotional bond with the organization. Thus, we argue that the positive effect of LMX on KSB via AC may be weaker for those individuals who are low in GSE. Taken together, we develop a moderated mediation model, in which high-quality LMX is positively and indirectly affect employees’ KSB via AC, with this indirect effect contingent on employees’ GSE. Thus, we propose the following:

*H3b*: Employees’ GSE moderates the mediated relationship of LMX with KSB through AC, such that the higher the level of GSE, the stronger the relationship.

#### The Negative Moderating Effect of ILOC on LMX-AC-KSB

Locus of control refers to the extent to which an individual believes that he/she can control his/her own fate (Rotter, 1966; Ng et al., 2006). Rotter (1966) differentiates this construct into two categories: ILOC and external locus of control (ELOC). Internal individuals usually believe that they can control over their fate and usually perceive a strong linkage between their behaviors and its consequences, whereas externals feel powerless and usually attribute what happens to them to factors beyond their control (Ng et al., 2006; Aubé et al., 2007). According to Ng et al. (2006) meta-analysis, ILOC shows positive effects on a wide range of work outcomes (e.g., well-being, motivation, and behavioral orientation). However, the current study will focus on ILOC’s negative effect: that is, high-ILOC people are relatively “immune” or not responsive to external reinforcement (Phares, 1965). These people believe that their personal traits such as talent and tenacity play more important roles in affecting their personal outcomes than external factors such as high-quality LMX.

High-quality LMX implies that favorable relationships between leaders and subordinates (e.g., getting support, praise, and recognition from leaders) are important factors in affecting subordinates’ outcomes (Casimir et al., 2014). This information disconfirms high-ILOC individuals’ belief that they can control over their personal outcomes. According to self-verification theory (Swann, 2011), high-ILOC individuals would neglect or be immune to positive information embedded in high-quality LMX, thereby attenuating the effect of LMX on their psychological reactions, such as AC, to the organization. In addition, individuals who have an ILOC feel they are able to control over their outcomes, they are likely to ascribe their rewards and punishment to their own actions rather than to the relationship with their leaders (Aubé et al., 2007). For example, they may consider their promotion as proof of personal ability rather than as an incentive from their leaders. Thus, ILOC may reduce the perception of gratitude and obligation to the organization, which weakens the positive effect of high-quality LMX on AC. In fact, previous studies demonstrate similar findings that positive external factors, such as leader consideration and charismatic leadership (e.g., Abdel-Halim, 1980; De Hoogh and Den Hartog, 2009), have less positive effects, and negative external factors, such as conflict and work stress (e.g., Krause and Stryker, 1984; Dijkstra et al., 2011), have less negative effects on high-ILOC individuals. Thus, we posit that employees with high ILOC are less attentive to the positive impact of high-quality LMX, rendering high-quality LMX less effective in promoting their AC.

In contrast, people who have an ELOC usually hold the belief that events are out of their control and put themselves in passive positions in regard to external environments (Ng et al., 2006). They are more sensitive to external factors and prefer to attribute personal outcomes to the environment or powerful others, such as their leaders (Chiu et al., 2005). In this regard, these people would pay more attention to high-quality LMX because they believe that their outcomes are dependent on these factors. Thus, when they feel that they are getting along well with their leaders, they are likely to show their gratitude toward the organization and to develop higher level of AC. At the empirical level, Chiu et al. (2005) study and Aubé et al. (2007) study both showed that ILOC weakens the positive relationship between leadership and AC whereas ELOC magnifies this relationship. Following these studies, we hypothesize that:

*H4a*: Employees’ ILOC moderates the positive relationship between LMX and AC, such that the higher the level of ILOC, the weaker the relationship.

Assuming that ILOC moderates the association between LMX and AC, it is also likely that ILOC will thus conditionally affect the indirect effects of LMX on KSB, just as in the theoretical assumption described in H3b, demonstrating a pattern of moderated mediation between these variables. As already explained, people high in ILOC are likely to attribute their outcomes to their own efforts, neglecting the external factors, such as high-quality LMX, that would substitute for the effect of high-quality LMX on AC. Because their emotional bonds with the organization are weak, their discretionary behaviors, such as KSB, will be not conspicuous. Conversely, low-ILOC individuals tend to pay more attention and react positively to high-quality LMX, making it more influential in strengthening their AC and KSB. In this regard, AC plays a more important mediating role in the LMX–KSB relationship. According to the above analysis, the following hypothesis is established:

*H4b*: Employees’ ILOC moderates the mediated relationship of LMX with KSB through AC, such that the higher the level of ILOC, the weaker the relationship.

## Research Methods

### Sample and Procedures

Data were collected from employees working in an information technology (IT) company in China. This organization is a medium-sized internet company which has about 1000 workers. Of the workers, 74% are male, 93% have a bachelor degree or above, and the average age are 32.4 years. There are about 40 project teams in this company. Each team has 1 or 2 team leaders and about 10–15 team members. These team leaders and members work together on specific tasks and they communicate frequently with each other. Thus, the supervisors know their subordinates’ behaviors well. The survey participants we selected were all from these project teams. Thus, this organizational context in our survey is suitable for exploring the relationship between LMX and KSB among employees.

We first asked our coordinators from this company to provide a list of supervisor–subordinate pairs. One team leader in a project team were asked to evaluate several team members. Before distributing questionnaires, we randomly assigned an identification number to a supervisor–subordinate pair, thus the supervisor’s evaluation could match with their subordinate’s response. In addition, the participants were informed that their participations were voluntary and anonymous, and the data was confidential. The coordinators distributed separate questionnaires to the supervisors and their subordinates. The supervisors needed to evaluate the KSB of their subordinates, and the subordinates needed to rate LMX, AC, GSE, and ILOC. In addition, the supervisors and subordinates were asked to fill the questionnaires in different places. When they finished rating, the completed questionnaires were returned in sealed envelopes. The coordinators distributed 300 sets of questionnaires. After a month, 231 completed questionnaires of matched supervisor–subordinate pairs were collected, for a response rate of 77%. The average age of supervisor sample was 35.2 years (SD = 7.34), and 81.4% were male. Some 93.5% had a bachelor’s degree or higher, and respondents had an average tenure with this company of 11.2 years (SD = 4.71). The subordinates sample had an average age of 29.2 years (SD = 5.47), and 68% were male. Some 93.1% had a bachelor’s degree or higher, and respondents had an average tenure with this company of 5.3 years (SD = 2.43).

### Measures

All measures were adopted from previously published papers. The Chinese version of the measures were developed by adopting back translation procedures. Unless otherwise informed, all items were rated on a five-point Liker-type scale with 1 indicating “strongly disagree” and 5 indicating “strongly agree.”

Leader-member exchange was measured using Graen and Uhl-Bien’s (1995) seven-item scale. This scale was used to evaluate the mutual respect between leaders and followers. A sample item for this scale was, “I have an excellent working relationship with my supervisor.” In the present study, the internal reliability was 0.87.

Items for measuring AC were adapted from Rhoades et al. (2001) six-item scale. This scale was used to assess the extent to which an employee is affectively committed to the organization. A sample item was, “I feel a strong sense of belonging to my organization.” In the present study, the internal reliability was 0.93.

General self-efficacy was measured using an eight-item scale developed by Chen et al. (2001). A sample item was, “I will be able to achieve most of the goals that I have set for myself.” In the present study, the internal reliability was 0.89.

We used an adapted version of a sixteen-item scale developed by Spector (1988) to assess employees’ levels of ILOC. This scale measures participants’ generalized control beliefs in their work outcomes, of which eight items were used to evaluate ILOC. We adopt these eight items to measure ILOC. A sample items was, “Most people are capable of doing their jobs well if they make the effort.” In the present study, the internal reliability was 0.83.

Subordinates’ KSB was evaluated by their immediate supervisors and measured using seven items developed by Lee et al. (2018). A sample item was, “The subordinate freely provides other members with hard-to-find knowledge or specialized skills.” In Lee et al.’s (2018) study, the coefficient alpha for this scale was 0.96. In the present study, the internal reliability was 0.90.

In line with previous recommendations (Kim et al., 2017), the demographic variables such as age, gender, education and tenure were used as controls in this study.

### Data Analysis

#### Measurement Model

Before testing the hypotheses, we first examined the convergent validity and discriminant validity of this model. The results (see Table 1) show that the factor loadings ranged from 0.71 to 0.87; the lowest average variance extracted (AVE) was 0.51; the lower limit of composite reliability (CR) was 0.88; and the Cronbach’s α of the scales were from 0.83 to 0.93. Moreover, the means, standard deviations, and intercorrelations of the studied variables are presented in Table 2. We can find that the square root of each construct’s AVE is greater than other correlation coefficients for the construct. Taken together, according to Fornell and Larcker (1981) suggestions, our model had acceptable convergent validity and discriminant validity.

**TABLE 1:** Convergent validity and reliability analysis.

**Constructs**	**Number of items**	**Factors loading range**	**Composite reliability (CR)**	**Average variance extracted (AVE)**	**Cronbach’s α**
LMX	7	0.71–0.81	0.90	0.53	0.87
AC	6	0.74–0.87	0.94	0.71	0.93
GSE	8	0.72–0.84	0.91	0.54	0.89
ILOC	8	0.71–0.78	0.88	0.51	0.83
KSB	7	0.78–0.84	0.93	0.64	0.90

**N* = 231.*

**TABLE 2:** Correlation between constructs.

**Variables**	**Mean**	**SD**	**AVE**	**1**	**2**	**3**	**4**	**5**
(1) LMX^a^	3.71	0.62	0.53	(0.73)				
(2) AC^a^	3.82	0.56	0.71	0.32^∗∗^	(0.84)			
(3) GSE^a^	3.97	0.89	0.54	0.25^∗∗^	0.29^∗∗^	(0.73)		
(4) ILOC^a^	3.83	0.73	0.51	0.27^∗∗^	0.28^∗∗^	0.69^∗∗^	(0.71)	
(5) KSB^b^	3.68	0.69	0.64	0.30^∗∗^	0.37^∗∗^	0.35^∗∗^	0.14^∗∗^	(0.80)

**N* = 231. ^∗∗^*p* < 0.01. Square roots of AVE are displayed on the diagonal in parentheses. ^*a*^These variables were measured from focal employees; ^*b*^managerial rating.*

#### Hypotheses Testing

The hierarchical regression results are shown in Table 3 (in this table “M” represents “Model”). Consistent with H1, the results show that LMX is positively related to KSB (M6; β = 0.27, *p* < 0.01). We adopted Baron and Kenny’s (1986) three-step method to test the mediating effect of AC. First, the result of the H1 show that the independent variable (i.e., LMX) significantly affect the dependent variable (i.e., KSB). Second, the results (Table 3, M2) show that LMX is positively related to AC (β = 0.33, *p* < 0.01). Finally, when both LMX and AC were entered into the regression model, the contribution of LMX became insignificant (M7; β = 0.06, *ns*), but the contribution of AC was significant (M7; β = 0.35, *p* < 0.01). Thus, the results suggested that the effect of LMX on KSB is fully mediated by AC.

**TABLE 3:** Hierarchical regression results.

**Variables**		**AC**				**KSB**						
		
		**M1**	**M2**	**M3**	**M4**	**M5**	**M6**	**M7**	**M8**	**M9**	**M10**	**M11**
Controls	Age	0.06	0.05	0.04	0.04	0.05	0.03	0.02	0.03	0.03	0.03	0.03
	Gender^a^	–0.03	–0.04	–0.02	–0.03	–0.02	–0.01	–0.02	–0.01	–0.02	–0.02	–0.02
	Education^b^	0.09	0.08	0.05	0.04	0.12^∗∗^	0.07	0.05	0.07	0.07	0.07	0.07
	Tenure	–0.04	–0.03	0.01	0.02	–0.01	–0.01	–0.03	–0.01	–0.01	–0.01	–0.01
IDV^*c*^	LMX		0.33^∗∗^	0.24^∗∗^	0.27^∗∗^		0.27^∗∗^	0.06				0.11^∗^
Mediator	AC							0.35^∗∗^	0.38^∗∗^	0.21^∗∗^	0.19^∗∗^	0.22^∗∗^
Moderator	GSE									0.24^∗∗^	0.27^∗∗^	0.25^∗∗^
	ILOC			0.16^∗∗^	0.11^∗∗^							0.07
Interaction	AC × GSE										0.22^∗∗^	0.09
Item	LMX × ILOC				–0.19^∗∗^							–0.17^∗∗^
	*R*^2^	0.02	0.11^∗∗^	0.18^∗∗^	0.28^∗∗^	0.04^∗^	0.12^∗∗^	0.17^∗∗^	0.15^∗∗^	0.22^∗∗^	0.28^∗∗^	0.31^∗∗^
	△*R*^2^		0.09^∗∗^	0.07^∗∗^	0.10^∗∗^		0.08^∗∗^	0.05^∗∗^	0.11^∗∗^	0.07^∗∗^	0.06^∗∗^	0.03^∗^

**N* = 231. M represents Model; ^*a*^Gender: male = 1, female = 0; ^*b*^Education: high school or less = 1, bachelor’s degree = 1, master’s degree or higher = 3; ^*c*^IDV, Independent variable; ^∗^*p* < 0.05; ^∗∗^*p* < 0.01.*

To further test the mediation effect, following Preacher and Hayes’s (2008) suggestion, a bias-corrected 95% confidence interval (CI) with 5,000 samples was conducted to test the significance of the estimated indirect effect. The bootstrapping results showed that the indirect effect of LMX on KSB via AC was significant (Estimate = 0.09, SE = 0.04, CI [0.03, 0.18]). Collectively, H2 was supported.

**FIGURE 2:**
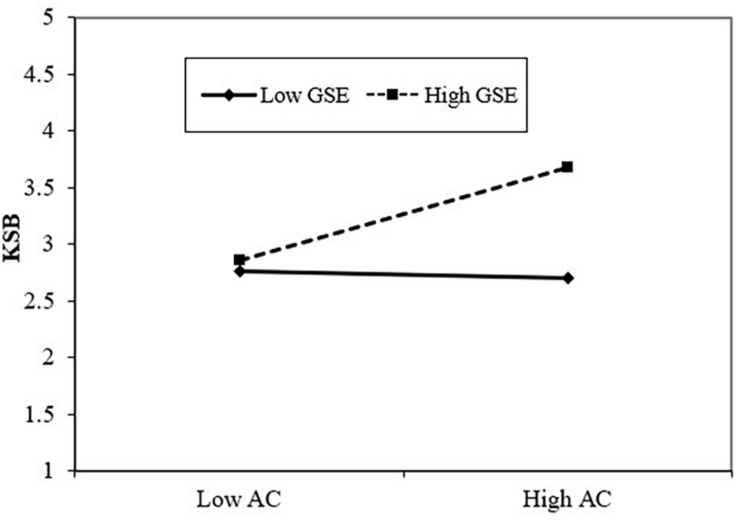
Interaction effect of AC and GSE on KSB.

To test the different moderating effect of GSE (H3a), we first mean-centered all the predictors to reduce multicollinearity (Aiken et al., 1991). Then KSB was regressed on the controls, AC, GSE and the interaction terms (AC × GSE). M10 of Table 3 shows that the interaction term (AC × GSE) was positively associate with KSB (β = 0.22, *p* < 0.01), suggesting that GSE magnified the positive effect of AC on KSB. Furthermore, in order to better understand the moderating effect, we plotted this moderating effect and conducted a simple slope test. The results (see Figure 2 and Table 4) showed that when GSE was high, AC was significantly related to KSB (*B* = 0.34, *p* < 0.01), whereas when GSE was low, the AC–KSB relationship was no longer significant (*B* = −0.04, *ns*). H3a is thus supported.

**TABLE 4:** Summary of the simple slope tests.

**Moderator levels**	***B***	**SE**	***t***	***p***
Low GSE	−0.04	0.03	0.88	0.381
High GSE	0.34	0.07	4.23	<0.001
Low ILOC	0.31	0.05	3.91	<0.001
High ILOC	0.03	0.02	0.76	0.449

*Low refers to one SD below the mean; High refers to one SD above the mean; SE refers to standard error.*

**FIGURE 3:**
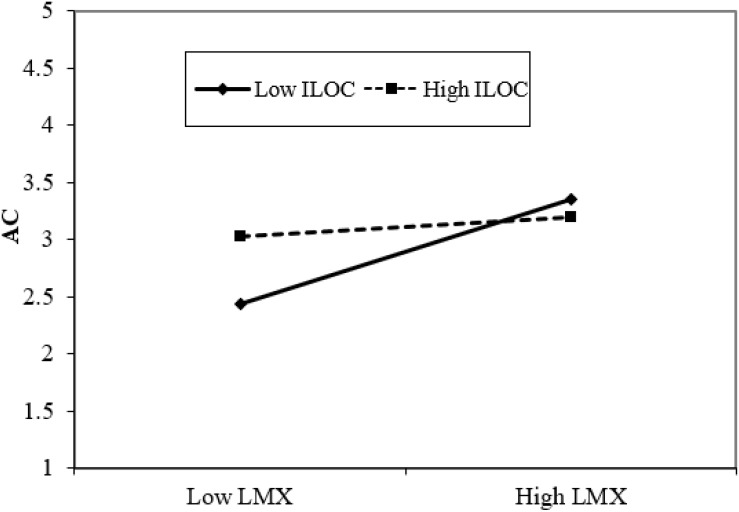
Interaction effect of LMX and ILOC on AC.

We adopted the same method to test H4a. AC was regressed on the controls, LMX, ILOC and the interaction terms (LMX × ILOC). M4 of Table 3 shows that the interaction term (LMX × ILOC) was negatively related to AC (β = −0.19, *p* < 0.01), revealing that ILOC attenuated the positive effect of LMX on AC. We also plotted this moderating effect and conducted a simple slope test. The results (see Figure 3 and Table 4) demonstrated that when ILOC was low, LMX was significantly related to KSB (*B* = 0.31, *p* < 0.01), whereas when ILOC was high, the LMX–AC relationship was no longer significant (*B* = 0.03, *ns*). Thus, H4a is supported.

We adopted Preacher et al. (2007) SPSS macro to examine the conditional indirect effects of LMX on KSB via AC at two values of the moderators (i.e., GSE and ILOC). We set high and low levels of the moderators at one SD above and below each moderator’s mean value, respectively. The results (see Table 5) show that the indirect effect of LMX on KSB via AC was conditional upon the levels of GSE and ILOC. The indirect effects were significant and stronger at a high level of GSE (Estimate = 0.21, SE = 0.06, CI [0.09, 0.45]) and a low level of ILOC (Estimate = 0.16, SE = 0.04, CI [0.06, 0.38]), but was insignificant and weaker at a low level of GSE (Estimate = −0.01, SE = 0.02, CI [−0.05, 0.02]) and a high level of ILOC (Estimate = 0.03, SE = 0.02, CI [−0.03, 0.07]). These results thus support H3b and H4b.

**TABLE 5:** Moderated mediation results for KSB across levels of GSE and ILOC.

**Moderator levels**	**Conditional indirect effect**	**SE**	**95% CI**	
			**Lower**	**Upper**
Low GSE	−0.01	0.02	−0.05	0.02
High GSE	0.21	0.06	0.09	0.45
Low ILOC	0.16	0.04	0.06	0.38
High ILOC	0.03	0.02	−0.03	0.07

*Low refers to one SD below the mean; High refers to one SD above the mean; SE refers to standard error; CI refers to confidence interval; Bootstrap sample size = 5,000.*

## Discussion and Conclusion

Knowledge sharing behavior allows organizations’ knowledge-based resources flow fluently and frequently, helps workers build on prior experience, and improves organizations’ contingency power (Hao et al., 2019); it is thus pivotal for organizational effectiveness and competitiveness. The exchange relationships between leaders and subordinates is considered an important source of determinants in predicting employees’ levels of KSB effort. Based on the person–situation interactionist perspective, the current study developed a moderated mediation model to explicitly answer the question of when and how LMX elevate subordinates’ KSB. Our results showed that AC fully mediates the positive relationship between LMX and KSB. Moreover, this mechanism is differently moderated by employee characteristics (i.e., GSE and ILOC). Specifically, GSE enhances the positive relationship between LMX and KSB via AC, whereas ILOC attenuates this mediating effect. These findings have implications for both theory and practice.

The theoretical contribution of this study is fourfold: First, although leadership has been considered a significant factor in affecting employee KSB, most prior papers have only emphasized the important role of leaders (e.g., Curtis and Taylor, 2018; Lee et al., 2018), neglecting the reactions of subordinates. They claim that various sorts of leadership can construct different climates in which employees exhibit different levels of KSB efforts. This argument may be unjustified. If followers are unable to develop comprehensive exchanges with their leaders, they may not accurately perceive these climates, resulting in markedly decreased effects. The current study emphasizes the LMX relationship, which not only consists of the behaviors of leaders but also highlights the reactions of subordinates. For instance, in high-quality LMX relationships, leaders are attentive to and supportive of their subordinates, while the subordinates are committed to and generate favorable attitude toward both the leaders and the organization (Dansereau et al., 1975). We argue that under this situation, employees will exhibit more extra-role behaviors, particularly KSB, for the organization. The result showed in Table 3 supports our assumption, showing a significantly positive relationship between high-quality LMX and KSB. In this regard, our study extends the current leadership–outcomes literature by (a) paying more attention to the reactions of followers and (b) adding new empirical evidence on the positive effects of LMX on various work outcomes.

Second, most previous studies assigned relatively little attention to the “black box” of the LMX–KSB relationship (Casimir et al., 2014; Kim et al., 2017). Our findings reveal that an employee’s emotional bond with the organization (i.e., AC to the organization) fully mediates the positive relationship between LMX and KSB, which offers a credible description of the above “black box.” While it has been suggested that high-quality LMX could elevate AC (Islam et al., 2013) and that employees’ emotional bonds with the organization could be an important antecedent in predicting KSB levels (Jeung et al., 2017), the current study introduces AC as a pivotal psychological mechanism (i.e., mediator) linking LMX to KSB. According to social exchange theory, the finding that LMX indirectly affects employees’ KSB via AC suggests that employees who develop strong AC to their organizations, induced by high-quality LMX, are inclined to participate in more extra-role behaviors, such as KSB, as a way of repaying the positive treatment they have received from their leaders. The above results can enhance our understanding of why high-quality-LMX employees contribute more to their organization than those with low-quality LMX. In addition, our study also reveals a direct positive association between AC and KSB. As far as we are aware, few studies have investigated psychological factors as determinants of KSB. Thus, why and when emotional and psychological factors determine KSB may provide a fertile ground for future research.

Third, although many researchers have highlighted the importance of the person–situation interactionist approach in studying employees’ work-related outcomes (e.g., Su et al., 2013; Zhou and Hoever, 2014; Hao et al., 2019), little research using this approach can be found in the KSB domain. Researchers of KSB (e.g., Seba et al., 2012; Papadopoulos et al., 2013; Marouf and Alrikabi, 2015) have predominantly chosen either an individual or a situational perspective, with few combining these two perspectives. The current study explicitly investigates the moderating role of employee characteristics (i.e., GSE and ILOC) in the indirect effect of LMX on KSB through AC. The findings reveal that the processes involved in transmitting high-quality LMX to KSB through AC seem to mainly improve the performance of employees who have high GSE and low ILOC. In effect, high-GSE individuals who also develop high AC to the organization induced by high-quality LMX, reap more benefits in terms of KSB, perhaps because their competence-oriented personalities have enabled them to pay attention to and react more actively to the optimal environment, increasing the likelihood of contributing more beneficial behaviors to the organization, such as KSB. However, high-ILOC employees who pay more attention to their own efforts may ignore the positive treatments received from high-quality LMX, decreasing the likelihood of building high levels of AC to the organization, which in turn results in less engagement in KSB. Thus, our study provides theoretical accounts and empirical evidence of how and why GSE and ILOC, two positive self-view constructs, show opposite moderating effects on the impact of high-quality LMX – a positive situation construct – on KSB through AC. In this regard, our findings advance the person–situation interactionist approach in KSB field, not only by offering new empirical results but also by delineating the different processes that produce different patterns of interactions.

Finally, the opposite moderating effects of GSE and ILOC demonstrated in our study shed new and nuanced light on the conceptual distinction between these two similar variables. In Judge’s and Bono’s meta-analysis, they found that self-esteem, GSE, ILOC, and emotional stability are all positively related to job-related outcomes (e.g., satisfaction and performance). They suggested that these positive self-concepts can construct a high-order variable to better predict job-related outcomes (Judge et al., 2003). Despite the simplification merit of this approach, it may lose sight of the nuanced differences among these traits. Some scholars have noticed this problem and found that GSE and self-esteem affect task performance via different motivational processes (Chen et al., 2004). In addition, De Hoogh and Den Hartog’s (2009) study showed that ILOC and emotional stability differently moderated the effects of leader behavior on burnout. The current study extends this line of research by applying self-verification theory to explicate the opposite self-verification processes regarding to the two similar elements of core self-evaluations (i.e., GSE and ILOC) and further reveals different moderating effects of these two traits. Thus, our study provides new theoretical insight into the conceptual difference between GSE and ILOC.

Our study also offers several useful practical implications. First, the quality of the relationships between leaders and subordinates could be an important determinant predicting employees’ voluntary behaviors (e.g., KSB). Nowadays, many organizations have invested in knowledge management systems; however, the effectiveness of their efforts could be tiny when the leaders and subordinates are experiencing low-quality relationships. The positive relationship between high-quality LMX and KSB suggests that more time and effort should be invested in training programs that can help both leaders and followers understand the importance of high-quality LMX and equip them with useful skills (e.g., social skills) to build good relationships with each other. In addition, the mediating effects of AC in the LMX–KSB relationship suggests that organizations should pay more attention to employees’ psychological mechanisms through which high-quality LMX elevates KSB. Thus, supervisors should take the initiative to perform some actions such as showing concern for subordinates’ feelings and needs, valuing their efforts and contributions, and creating ongoing informative feedback for them to enhance their AC to the organization.

The contrasting moderating effects of GSE and ILOC in the LMX–AC–KSB relationship suggest that managers should build flexible relationships with their followers who have different self-evaluations. Managers should be trained to discern the level of GSE and ILOC of their followers by observing their daily behaviors. Moreover, systematic personality tests should be conducted to better understand the subordinates’ levels of GSE and ILOC. Such information can help managers decide how to develop different relationships with different subordinates, so that high-quality LMX can maximally facilitate KSB. For employees who consider themselves efficacious, managers should communicate with them clearly and frequently to confirm their mastery self-view, enhancing their desire to exhibit KSB. With respect to high-ILOC employees, the leader’s role in affecting a subordinate’s outcomes within a high-quality LMX relationship should be downplayed so that these people do not feel a loss of personal control.

Our study also has some no limitations. First, ratings for LMX, AC, GSE, and ILOC were collected from the same source (i.e., employees). Although we tried to minimize the common method bias and enhance the objectivity of the data by measuring KSB using a different source (i.e., supervisors), these problems still cannot be entirely ruled out. For example, in the Chinese culture, *guanxi* is an important factor affects how followers exchange with their leaders (Wang et al., 2012). Many Chinese workers may focus more on developing “upward” relationships with their leaders and be less willing to invest in “downward” associations with their subordinates (Kim et al., 2015). In this respect, measuring LMX solely from the perspective of subordinates may cause bias. Future studies should then complement subordinate-assessed LMX with supervisor ratings, as well as supervisor–subordinate agreement on LMX. Second, our study adopts a cross-sectional research design which may prevent us from explaining the determinations of causality among the variables explicitly. Conducting a longitudinal study or experimental study can provide stronger evidence for the causal relationships in the proposed model. For example, it would be interesting to investigate whether LMX quality changes over time and how this change affects employees’ AC and KSB. Third, our data were collected from a single IT company in a single cultural context. This sample may hinder the generalizability of our findings to other fields in other cultural contexts (e.g., Western societies). Therefore, we would advocate replicated studies that use data from multiple organizations with different job types in different cultural contexts in the future. Furthermore, because our study is focused, many other personal characteristics and organizational factors that may influence the key variables in our study are not incorporated. Adopting other individual factors such as exchange ideology (Kim et al., 2017) and other organizational aspects such as organizational justice (Lee et al., 2018) as moderators might be encouraged in future studies.

## Data Availability Statement

The datasets generated for this study will not be made publicly available. The datasets are available only with the permission of the surveyed organization.

## Ethics Statement

The studies involving human participants were reviewed and approved by the Institutional Review Board (IRB) of the School of Labor and Human Resources, Renmin University of China with written informed consent from all subjects. All subjects gave written informed consent in accordance with the Declaration of Helsinki.

## Author Contributions

All authors listed have made a substantial, direct and intellectual contribution to the work, and approved it for publication.

## Conflict of Interest

The authors declare that the research was conducted in the absence of any commercial or financial relationships that could be construed as a potential conflict of interest.
